# Case Report: Appearance of Various Disease-Specific Antibodies After the Onset of Dipeptidyl Peptidase-4 Inhibitor-Associated Bullous Pemphigoid

**DOI:** 10.3389/fimmu.2022.843480

**Published:** 2022-03-03

**Authors:** Yuichiro Iwamoto, Takatoshi Anno, Katsumasa Koyama, Fumiko Kawasaki, Kohei Kaku, Koichi Tomoda, Seiko Sugiyama, Yumi Aoyama, Hideaki Kaneto

**Affiliations:** ^1^Department of General Internal Medicine 1, Kawasaki Medical School, Okayama, Japan; ^2^Department of Dermatology, Kawasaki Medical School, Okayama, Japan; ^3^Department of Diabetes, Endocrinology and Metabolism, Kawasaki Medical School, Kurashiki, Japan

**Keywords:** dipeptidyl peptidase-4 inhibitor-associated bullous pemphigoid, dipeptidyl peptidase-4 inhibitor, autoantibodies, type 2 diabetes mellitus, bullous pemphigoid

## Abstract

Bullous pemphigoid (BP) is a rare autoimmune blistering disease, and the prevalence of type 2 diabetes mellitus (T2DM) is relatively high in subjects with BP. It is known that dipeptidyl peptidase-4 inhibitor (DPP-4i), one kind of antidiabetic drugs, can cause BP, although precise mechanism of DPP-4i-related BP remains unclear. In this report, we showed a case with appearance of various disease-specific antibodies after the onset of DPP-4i-related BP. Furthermore, various disease-specific antibodies became positive and showed high titers two years after the onset of DPP-4i-related BP and discontinuation of DPP-4i. These data showed that it is possible for immune tolerance to be broken after the onset of DPP-4i-related BP, and it may be important to check autoimmune antibodies in DPP-4i-related BP subjects even when BP symptoms are improved.

## Introduction

Various pathological autoantibodies are produced in subjects with various immune system disorders, which recognize some body’s normal constituent as “self”. However, there are various causes of autoantibody production, and its precise mechanism is not well understood. Bullous pemphigoid (BP) is a rare autoimmune blistering disease, but its incidence has been increasing. Previously, it was reported that the prevalence of type 2 diabetes mellitus (T2DM) was relatively high in subjects with BP in the last decade (1, 2). It has been hypothesized that T2DM may represent a disease of the innate immune system responsible for an ongoing cytokine-mediated acute phase response (3). In addition, it is known that dipeptidyl peptidase-4 inhibitor (DPP-4i), one kind of antidiabetic drugs, can cause BP (4), although precise mechanism of DPP-4i-related BP remains unclear.

In this report, we show a subject with DPP-4i-related BP in which various autoantibodies were induced one after another after the onset of DPP-4i-related BP. Furthermore, titers of such various disease-specific antibodies were continuously increased, although they did not lead to the onset of various diseases.

## Case Description

**Figure 1:**
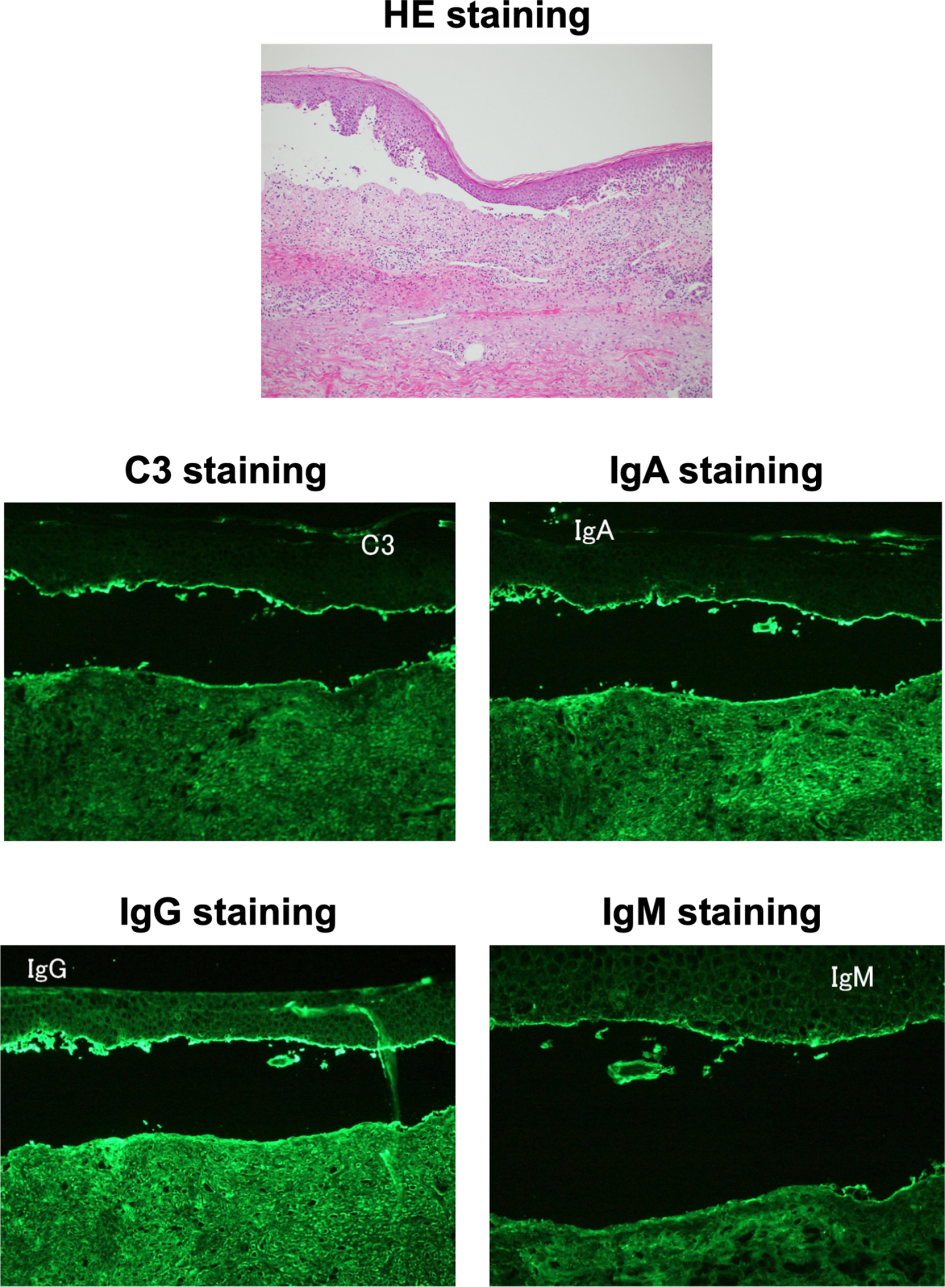
Histopathological microscopic findings on skin biopsy. The hematoxylin and eosin staining of the skin biopsy (upper panel) showed a sub-epidermal cleavage. C3, Immunoglobulin (Ig) G, IgA and IgM staining (middle and lower panels) showed linear deposit at the membrane zone, respectively.

An 85-year-old Japanese man was referred to our hospital for BP. He was diagnosed as T2DM at age of 65, and he started taking 20 mg/day of teneligliptin at age of 84 and 100 mg/day of vildagliptin at age of 85. After then, he had symptoms of tense blisters over erythematous skin on dorsal surface of the hand. Diffuse bullous eruption with tense blisters was observed from the forearm to upper arm. The drug lymphocyte stimulation test (DLST) showed positive for vildagliptin (30647 cpm, control 744 cpm). Anti-BP180-NC16a antibody, an autoimmune marker of BP, was elevated to 31.5 U/mL. We performed a skin biopsy and direct and indirect immunofluorescence assays. As shown in **Figure 1**, a sub-epidermal cleavage was observed. In addition, linear deposit of C3, Immunoglobulin (Ig) G, IgA and IgM was observed at the membrane zone. Based on these findings, we diagnosed this subject as DPP-4i-related BP. Autoantibody-associated data were as follows: anti-double stranded DNA IgG (dsDNA) antibody was positive (17 IU/mL), whereas anti-nuclear antibody (ANA), anti-ribonucleoprotein (RNP) antibody, anti-Sm antibody, anti-SS-A/Ro antibody and anti-SS-B/La antibody, anti-glutamic acid decarboxylase (GAD) antibody were all negative. He did not have symptoms of collagen and mucosal lesions (including BP-related mucosal change), although dsDNA antibody showed positive.

On admission, we stopped DPP-4i, and he was treated with insulin therapy, since his glycemic control was poor. His symptoms of tense blisters and erythematous skin were improved with topical steroids therapy (clobetasol propionate), although systemic steroid therapy by oral administration or injection is employed in some cases. At that time, platelet level was decreased to 8.7×10^4^/μL, and one month later, it was further decreased to 5.1×10^4^/μL. We examined the possible cause of idiopathic thrombocytopenic purpura (ITP). Platelet-associated IgG (PAIgG) was elevated to 78 ng/10^7^cells (positive) and anti-platelet antibody was negative. He was discharged after improvement of DPP-4i-related BP and treated with other antidiabetic drugs except of DPP-4i.

**Figure 2:**
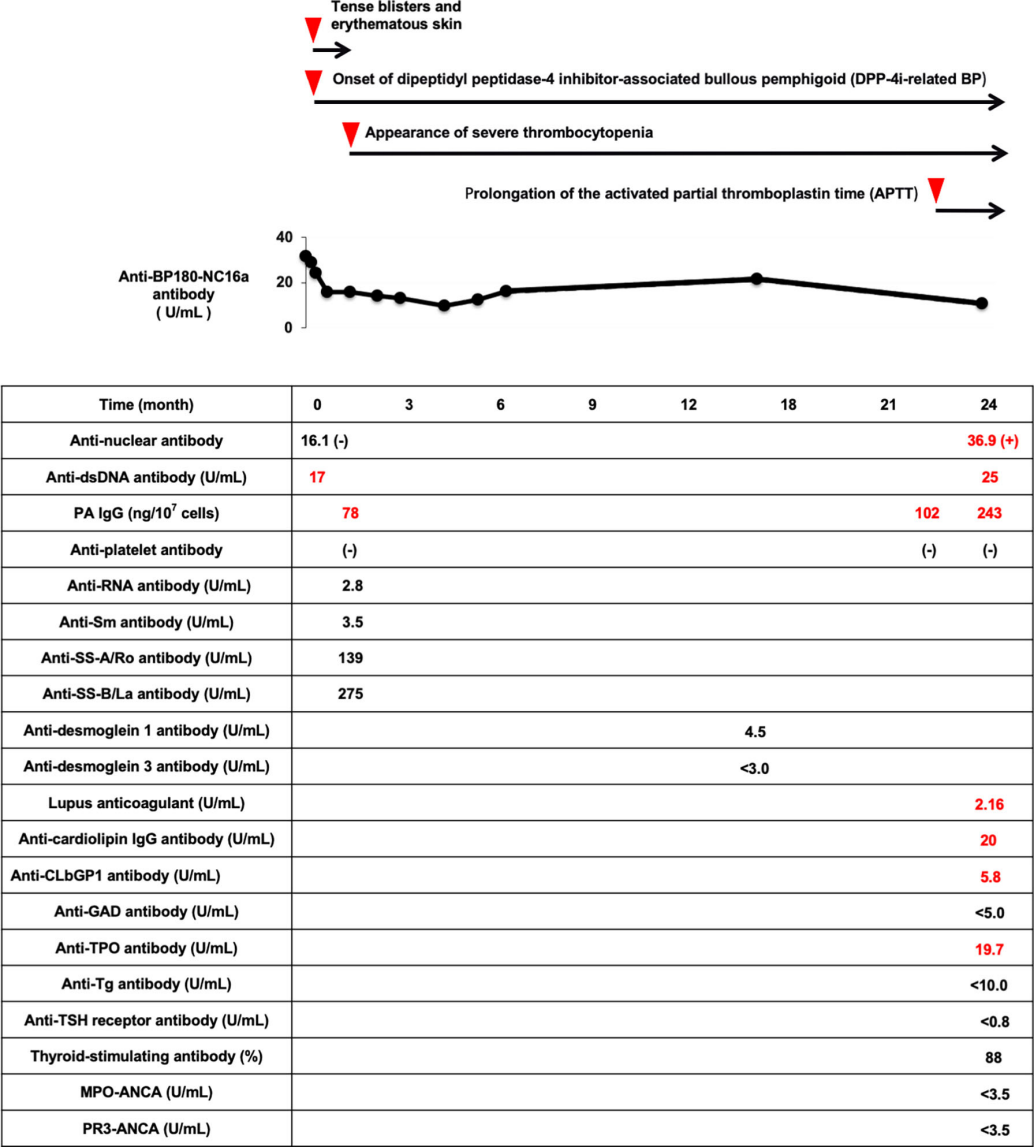
Time course of the appearance of various disease-specific antibodies after the onset of DPP-4i-related BP in this subject. After the onset of DPP-4i-related BP, various disease-specific antibodies are continuously produced and their titers are elevated. DPP-4i-related BP, dipeptidyl peptidase-4 inhibitor-associated bullous pemphigoid; APTT, activated partial thromboplastin time; ANA, anti-nuclear antibody; dsDNA, double stranded DNA IgG; PAIgG, platelet-associated IgG; RNP, ribonucleoprotein; GAD, glutamic acid decarboxylase; CLβ2GP1, cardiolipinβ2-glycoprotein I; TPO, thyroid peroxidase; Tg, thyroglobulin; MPO-ANCA, myeloperoxidase-anti-neutrophil cytoplasmic antibody; PR3-ANCA, neutrophil cytoplasmic antibody.

After discharge, his symptoms of DPP-4i-related BP were stable, although he sometimes had tense blisters and used topical steroids therapy. Chronic low platelet level was continuously observed. Since he had no symptoms such as bleeding tendency, we observed the thrombocytopenia. Anti-BP180-NC16a antibody was continuously positive (9.8-16.2 U/mL), but anti-desmoglein 1 and 3 antibodies were both negative. When he suffered from pneumonia at age of 87, he had prolongation of the activated partial thromboplastin time (APTT). We examined various autoantibodies again. Anti-dsDNA antibody was elevated to 25 IU/mL (positive) and ANA was positive (41.3). In addition, lupus anticoagulant (2.16), anti-cardiolipin immunoglobulin G antibody (20 U/mL) and anti-cardiolipinβ2-glycoprotein I antibody (5.8 U/mL) were all positive. Myeloperoxidase-anti-neutrophil cytoplasmic antibody (MPO-ANCA) and proteinase3-anti-neutrophil cytoplasmic antibody (PR3-ANCA) were negative. Anti-thyroid peroxidase antibody was slightly positive (5.8 U/mL), although anti-thyroglobulin antibody and thyrotropin receptor antibody were both negative. Moreover, PAIgG was increased to 276 ng/10^7^cells (positive). Although his various autoantibodies became positive and showed high titers two years after the onset of DPP-4i-related BP, we observed him in consideration of his old age and lack of clinical symptoms. **Figure 2** shows a time course of various disease-specific antibodies after the onset of DPP-4i-related BP in this subject. After the onset of DPP-4i-related BP, various disease-specific antibodies were continuously produced and their titers were elevated.

## Discussion

Herein, we report a case of DPP-4i-related BP, and interestingly his various disease-specific antibodies became positive after the onset of DPP-4i-related BP. It has been shown that the frequency of DM is relatively high in subjects with BP. Furthermore, it is considered that such high frequency of DM in BP patients is, at least in part, due to the increased usage of DPP-4i. In the case of drug-induced BP, the most important treatment is the withdrawal of the culprit drug.

DPP-4i-related BP is an autoimmune blistering disease. Although early immature B-cells have potential to produce various autoantibodies associated with autoimmunity even in healthy donors, mature memory B-cells do not normally produce detectable levels of autoantibodies under normal conditions (5). However, once some environmental factors, such as infection or hyperglycemia, are added to subjects under immune tolerance conditions, the immune system of B-cell tolerance is broken and ultimately results in activating self-reactive lymphocytes and production of antibody-secreting plasma cells in these patients (6).

DPP-4, also known as a cluster of differentiation 26 (CD26) molecule, is characterized as a T-cell differentiation antigen and is a cell surface glycoprotein expressed in various types of cells and tissues. DPP-4 plays a multifunctional role through its enzymatic and nonenzymatic action. However, the immune regulatory function of DPP-4 in various types of cells and tissues remains poorly characterized and the interaction of the DPP-4 molecule and the immune system is complex. In cutaneous pathology, it was revealed that DPP-4 plays a significant role in BP, T helper type 1-like reactions, cutaneous lymphoma, melanoma, wound healing and fibrotic disorders. DPP-4 modulates the immune response in a highly complex manner through its actions on T-cell survival, activation and T-cell-mediated increase in pro-inflammatory cytokines, along with the induction of skin-specific lymphocytes. DPP-4-mediated imbalance in the homeostasis of regulatory and effector T cells may aggravate the pathophysiology of autoimmune/autoinflammatory dermatoses (7). Therefore, there are some concerns that DPP-4i may affect some part of the immune system (8), and thus DPP-4i has the potential to modulate various immunological functions. Although there are some hypotheses about the onset of DPP-4i-related BP (9), precise mechanism for DPP-4i-related BP remains unclear. In addition, it is possible that some of additional factors lead to the onset of DPP-4i-related BP as well as the onset of autoimmune disease. Very interestingly, the usage of DPP-4i in our patient caused not only BP but also facilitated the promotion of other antibodies. These data suggest that once DPP-4i-related BP is brought about, there is a possibility that various other autoimmune-diseases are also induced.

Recently, several studies have evaluated the association of different DPP-4i with overall autoimmune diseases directly, although age as an important determining factor of autoimmunity is associated with DPP-4i use (10–14). In our case, he was treated with DPP-4i for T2DM until the onset of DPP-4i-related BP. However, he was not treated with DPP-4i after the onset of DPP-4i-related BP and during the appearance of various disease-specific antibodies. Therefore, we think that the appearance of various disease-specific antibodies was associated with broken immune system, which led to facilitate self-reactive production of various antibodies after the onset of DPP-4i-related BP. Once his immune system was broken with DPP-4i and/or DPP-4i-related BP, broken immune system tolerance was continuously observed even after stopping DPP-4i due to the appearance of various disease-specific antibodies.

There is a limitation in this case reports. First, it was possible that appearance of various disease-specific antibodies was just coincidence because of higher age. BP is a blistering skin condition that occurs commonly in the elderly population, and aging is one of the risk factors for autoimmune diseases (15). On the other hand, BP itself is associated with autoimmune disease (16). Since our patient had the appearance of various disease-specific antibodies after the onset of DPP-4i-related BP, some disruption of immune tolerance induced by DPP-4i-related BP, at least in part, affected the appearance of other disease-specific antibodies. Second, some of his disease-specific antibodies showed low titer, and all of disease-specific antibodies may not be in all cases of significance altogether at these low levels. However, many of these disease-specific antibodies newly appeared or gradually increased in this case in which anti-BP180-NC16a antibody showed positive continuously although the titer was low. Therefore, we thought that we had to pay attention to other disease-specific antibodies during the time course, although it is necessary to keep in mind cost-benefit aspect.

## Conclusion

We should bear in mind that autoimmune antibodies can appear after the onset of DPP-4i-related BP. In such cases, various disease-specific antibodies are continuously produced and their titers are elevated. Therefore, it would be important to repeatedly check autoimmune-antibodies in DPP-4i-related BP subjects even when BP symptoms are improved.

## Data Availability Statement

The original contributions presented in the study are included in the article/supplementary material. Further inquiries can be directed to the corresponding author.

## Ethics Statement

Written informed consent was obtained from the individual(s) for the publication of any potentially identifiable images or data included in this article.

## Author Contributions

YI and TA researched data and wrote the manuscript. KaK, FK, and SS researched data and contributed to the discussion. KoK, KT, YA, and HK reviewed the manuscript. All authors contributed to the article and approved the submitted version.

## Conflict of Interest

The authors declare that the research was conducted in the absence of any commercial or financial relationships that could be construed as a potential conflict of interest.

## Publisher’s Note

All claims expressed in this article are solely those of the authors and do not necessarily represent those of their affiliated organizations, or those of the publisher, the editors and the reviewers. Any product that may be evaluated in this article, or claim that may be made by its manufacturer, is not guaranteed or endorsed by the publisher.
